# Differences in Dietary Patterns among the Polish Elderly: A Challenge for Public Health

**DOI:** 10.3390/nu13113966

**Published:** 2021-11-07

**Authors:** Robert Gajda, Marzena Jeżewska-Zychowicz, Ewa Raczkowska

**Affiliations:** 1 Department of Human Nutrition, Faculty of Biotechnology and Food Sciences, Wrocław University of Environmental and Life Science, Norwida 25, 50-375 Wroclaw, Poland; ewa.raczkowska@upwr.edu.pl; 2 Department of Food Market and Consumer Research, Institute of Human Nutrition Sciences, Warsaw University of Life Sciences (SGGW-WULS), Nowoursynowska 159C, 02-776 Warsaw, Poland; marzena_jezewska_zychowicz@sggw.edu.pl

**Keywords:** the elderly, dietary patterns, socio-economic status, family, region

## Abstract

The aim of the study was to assess the diversity of dietary patterns within the elderly, in relation to the region of residence, household structure, and socioeconomic status. The questionnaire was conducted in a group of 427 Polish adults aged 60 and older from June to September 2019. The sample was selected by means of the snowball method in two regions. Principal component analysis (PCA) was used to extract and identify three dietary patterns (factors) from the frequency of eating 32 groups of foods. Logistic regression analysis was used to determine the relationship between the identified dietary patterns (DPs), region, household status, and socioeconomic index (SES). Adherence to the identified DPs, i.e., traditional, prudent, and adverse, was associated with socioeconomic status (SES) and living environment, i.e., living alone, with partner, or with family, while the region did not differentiate them. Less people living with their family were characterized by the frequent consumption of traditional food (the upper tertile of this DP), while more of them often consumed food that was typical for both prudent and adverse DPs (the upper tertiles of these DPs). The presence of a partner when living with family did not differentiate the adherence to DPs. A high SES decreased the chances of adhering to the upper tertiles of the “prudent” and “traditional” DPs, while living with family increased the chances of adhering to both the upper and middle tertiles of the “prudent” DP. Identifying the dietary patterns of the elderly contributes to a better understanding of the food intake of the senior citizens living in different social situations, in order to support public policies and nutritional counseling among this age group.

## 1. Introduction

Ageing is associated with a progressive physical and cognitive decline. Therefore, adequate nutritional intake is very important in the elderly [1,2] (inadequate nutrition can lead to various dysfunctions, such as decreased immunity, frailty, and noncommunicable diseases (NCDs)). Overweight and obesity significantly increase the risks of these dysfunctions [3]. The most common dietary mistakes in the elderly include the following: poor variety of meals; insufficient intake of vegetables and fruit, dairy products, cereals, fish, and water; excessive intake of sugar and sweets, meat and its products, fats, and foods with high energy density and low nutrient density [4].

Recent approaches to studying health-related behaviors have adopted dietary patterns, rather than investigating individual exposures, i.e., food intake and/or macronutrient intake [5]. Dietary patterns (DPs) represent the whole diet and are considered to be a better alternative to single dietary characteristics when studying the associations between diet and other variables, i.e., chronic diseases and food choice motives [6,7]. To identify DPs, factor analysis (FA) and principle component analysis (PCA) can be applied [7]. The aggregation of dietary data, e.g., frequency of food consumption, into factors based on correlations between input variables allows the identification of DPs that express the specificity of the diet of the study group.

The dietary patterns that were often identified in previous research are “prudent” and “western” [8,9,10]. The “traditional” DP was also often distinguished, i.e., dietary pattern specific for the country in which the research is conducted [11,12]; for example, the “traditional Polish” DP usually consists of potatoes, meat, vegetables, cheese, animal fats, and sugar, which are foods typical of traditional Polish cuisine [12]. Up until now, studies have distinguished dietary patterns among the elderly that are more beneficial to health, such as “prudent”, “healthy”, or “Mediterranean”, those that are more detrimental to health, i.e., “western” or “unhealthy” DPs [13,14,15,16,17], as well as a “traditional” dietary pattern [15,18].

The “traditional DP”, however, has some internal differences within it. A number of studies confirm variations in dietary patterns resulting from the regional culture that exists within national traditions and heritage [19,20]. Research on the regional differences in DPs, including the traditional DP, is sparse in Poland. Nevertheless, in the study by Czarnocińska et al. [21], carried out in young women, the differences in DPs between regions were confirmed. A stronger relationship between dietary patterns and region than that between DPs and family socioeconomic status was revealed in a representative sample of Polish young women. However, there were differences based on socioeconomic status in the regions; for example, the ‘fruit and vegetables’ pattern was observed in the more affluent regions, and also in families with a high SES index, while the ‘traditional Polish’ pattern showed no regional differences, but it was less common among the families with a higher socioeconomic status. This suggests that the regions within the country may become effective focus areas for specific dietary interventions. Such a relationship can be expected among older people as well, but, in this age group, other factors can also have a strong differential effect on dietary patterns, i.e., socio-economic status, family status, and health status [22,23,24].

Regional differences in DPs may originate from cultural and social environments (e.g., family status), but they can also root from economic status [25]. The importance of socioeconomic status (SES) in the differentiation of dietary patterns was confirmed in previous studies [20,26]. Generally, a higher socioeconomic status is associated with healthier food choices [25,27]. However, such relationships are stronger in higher-income countries than in lower–middle- or lower-income countries [28]. Mayén et al. [27] observed that in middle- and low-income countries, people with a higher SES may display more varied eating behavior, e.g., they display healthy behaviors, as well as having a higher intake of energy, cholesterol, or saturated fatty acids; for example, those with a higher income and higher education favored the dietary pattern called “ultra-processed food” [29]. People in low-income countries are more likely to have “unhealthy” eating patterns regardless of the SES level [20]. Hence, the varied and inconsistent results on the relationship between dietary patterns and socioeconomic status require further research, and especially investigations that take regional differences into account.

Previous studies have shown better health of the elderly living with other people in comparison with those living alone [22,30,31]. There are also differences depending on who lives with the elderly in the household [32,33]. However, the results are often inconclusive, and they are related to food intake and/or macronutrient intake, rather than to dietary patterns [34,35,36]. Although these studies are scarce, their results indicate a worse quality of the diet of elderly people living alone, compared to those living with their families [23,24].

Although studies of dietary patterns have recently gained a lot of interest, most of them are carried out among young people and adults, while the elderly are included to a lesser extent. Moreover, most of the research focused on the relationship between DPs and the region of residence and socioeconomic status, which mainly concerns young people [21] or adults [19,20,26,27]. There is a lack of research on the relationship between the structure of the household of the elderly and their dietary patterns, while food intake and diet quality have been studied more intensively [24,34,35]. Moreover, these studies mainly concerned countries other than Poland. Therefore, the aim of the study was to identify the dietary patterns of Polish seniors, and then to assess the diversity of these patterns with regard to the region of residence, household structure, and socioeconomic status.

## 2. Material and Methods

### 2.1. Study Design and Sample

The research was carried out in two culturally and economically diverse regions in Poland. In 2019, the Świętokrzyskie region was the region with the lowest GDP (71.6% of medium GDP per capita), while the Śląskie/Dolnośląskie region, including two voivodeships, i.e., Śląskie and Dolnośląskie, was characterized by high GDP (102.3% and 109.5% of medium GDP per capita, respectively) [37]. The study was conducted from June to September 2019. The sample was selected using the snowball method. A total of 750 questionnaires were distributed in 16 clubs or senior circles in both regions. People who agreed to participate in the study were asked to help in further recruitment by handing over a questionnaire to people living in their neighborhood who met the age criterion. As a result, 506 questionnaires were collected. Due to the lack of data, 69 questionnaires were excluded from the analysis. The inclusion criterion for the study was the age of 60 years or more, and that each participant represented one household. The study sample consisted of 437 people, with 251 participants from the Śląskie/Dolnośląskie region and 186 participants from the Świętokrzyskie region.

### 2.2. Questionnaire

A dietary habits and nutrition beliefs questionnaire (KomPAN) [38,39] was used to assess the frequency of consumption of 32 groups of foods. All participants were asked to record their habitual frequency of consumption for each food group within the last year using the following answers: (1)—less than once a month or never; (2)—1–3 times a month; (3)—once a week; (4)—a few times a week; (5)—once a day; (6)—a few times a day.

To assess the household structure, the question “What is your household composition?” was posed with the following answers: (1)—I live alone; (2)—I live with a partner; (3)—I live with family without a partner; (4)—I live with family and a partner. The questions on sociodemographic characteristics of the study group concerned gender, age, education, and place of residence.

To assess the socio-economic status (SES) of the respondent, the following questions were asked:

1. Self-reported financial situation—the following two questions addressed this matter: “How do you assess your financial situation?”, with the following answers: below average (1 point); average (2 points); above average (3 points), and “*How do you evaluate the situation of your household?*”, with the following answers: I have to save to meet my basic needs (1 point); it is enough for my needs, but I have to save for larger purchases (2 points); it is enough for me without saving (3 points).
2. Family financial assistance—the following question was asked: “Do you obtain financial assistance from your family including the family you live with?”, with the following answers: no, although I have financial problems (1 point); yes, because I have financial problems (2 points); there is no such need because my financial situation is satisfactory (3 points); yes, although I have no financial problems (4 points).
3. Social financial assistance was addressed by the question “Do you obtain social assistance related to finances?”, with the following answers: no, although I have financial problems (1 point); yes, because I have financial problems (2 points); there is no such need because my financial situation is satisfactory (3 points); yes, although I have no financial problems (4 points).
4. Education—the following question was asked: “What is your education?”, with the following answers: primary (1 point); vocational (2 points); secondary (3 points); higher education (4 points).

The socioeconomic status (SES) of the elderly was calculated using a procedure similar to the previously developed SES index [40,41]. The SES index was calculated for each participant by summing up the points for each variable, i.e., self-reported financial situation, family and social assistance and education. To assess the reliability of the input data of the SES index, Cronbach’s alpha index was used [42]. The Cronbach’s alpha coefficient for the variables included in the SES index was 0.683. Groups of participants with low, medium and high SES indexes were distinguished on the basis of the tertile distribution of the SES index.

### 2.3. Statistical Analysis

Qualitative variables are presented as percentages (%). The chi-square test was used to verify the differences between those variables. Principal component analysis (PCA) was used to extract and identify dietary patterns from the frequency of eating 32 groups of foods. As a result, three factors (dietary patterns—DPs) were distinguished. The factors were rotated by Varimax transformation. The number of identified factors was based on the following criteria: components with an eigenvalue of 1, a scree plot test, and the interpretability of the factors. Food items were considered to load on a factor if they had a correlation of a minimum of 0.5 with it. The factorability of data was confirmed with the Kaiser—Meyer—Olkin (KMO) measure of sampling adequacy and Bartlett’s test of sphericity, both of which achieved statistical significance. The KMO value was 0.798. Bartlett’s test had a significance of *p* < 0.0001. In each DP, three categories (tertiles) were identified, which are described as bottom (1st), middle (2nd) and upper (3rd) tertiles.

Logistic regression analysis was used to assess the relationship between identified dietary patterns (DPs), region, household structure, and socioeconomic index (SES). Odds ratio (OR) values were calculated at the 95% confidence level. The reference group (OR = 1.00) was the bottom tertile of all DPs. A p-value lower than 0.05 was considered significant for all tests. Statistical analysis was performed using the STATISTICA statistical software (version 13.3, PL; StatSoft Inc., Tulsa, OK, USA; StatSoft, Kraków, Poland).

## 3. Results

### 3.1. Characteristics of the Study Sample

Table 1 displays the socio-demographic characteristics of the study sample. In this sample, 66.1% were women and 33.9% were men. People aged 60–74 accounted for three fourths of the sample. The biggest group of respondents lived in the countryside (46.2%). Almost 60% of the respondents lived in the Śląskie/Donośląskie region. More than 2/5 of the respondents lived with a partner, and over a quarter lived with a partner and family. The proportion of respondents characterized by low, medium and high SES was similar, at about 1/3 for each SES level.

### 3.2. Dietary Patterns

Table 2 illustrates the correlations between the frequency of eating various food groups and each of the identified DPs. The DPs were categorized into the “traditional” DP (factor 1), the “prudent” DP (factor 2), and the “adverse” DP (factor 3). The “traditional” DP was characterized by a high frequency of eating white bread and bakery products, fried foods, cold meats, smoked sausages, hot dogs, and potatoes. The “prudent” DP was characterized by a high frequency of eating buckwheat, oats, wholegrain pasta or other coarse-ground groats, fermented milk beverages, fresh cheese curd products, fruit, vegetables, vegetable juices or fruit and vegetable juice, and water. The “adverse” DP was characterized by a high frequency of consuming sweetened carbonated or still beverages, energy drinks, instant soups or ready-made soups, tinned (jar) meats, and lard.

The characteristics of DPs, with regard to the region, SES index, and household structure, are presented in Table 3. There were no differences in DPs with regard to the region. Less respondents who lived without a partner, but with their family, were characterized by the frequent consumption of Polish traditional food (the upper tertile of the “traditional” DP), while the frequent consumption of food characteristic for the other dietary patterns, i.e., “prudent” and “adverse” DPs, was observed in this group. Similar characteristics concerned people living with a partner and a family. However, in the “adverse” DP, there were no differences in the number of people in the bottom and upper tertiles (33.6% and 34.5%, respectively). More people with a low SES were in the upper tertiles of the “traditional” (44.7%) and “prudent” (50.0%) DPs, and also in the bottom tertile of the “adverse” DP (36.8%); however, the differences were not statistically significant. On the other hand, the biggest percentage of people with a high SES were in the bottom tertiles of the “traditional” (41.0%) and “prudent” (48.9%) DPs, and also in the upper tertile of the “adverse” (36.7%) DP. The same relationship was shown for only two variables forming the SES. The highest proportions of people describing their financial situation with the phrase “above average” were in the lower tercile of the “traditional” DP (21.5%) and the “prudent” DP (20.6%), and in the upper tercile of the “adverse” DP (14.4%). Furthermore, the highest percentage of people with higher education was in the upper tercile of the “traditional” DP (22.8%) and the “prudent” DP (30.1), and in the upper tercile of the “adverse” DP (21.4) (Table 3).

The results of the logistic regression have demonstrated that the respondents who consumed healthy food most often (the upper tertile of “prudent” DP) were 1.6 times more likely to live without a partner, but with their family. Moreover, they were two times more likely to live with a partner and with their family. The people who represented the upper tertile of the “traditional” and “prudent” DPs were less likely to have a high SES (Table 4).

## 4. Discussion

Our study has identified three dietary patterns, which is in the range of the factors distinguished in other studies [43]. The identified dietary patterns are as follows: “traditional”, “prudent”, and “adverse”. Similar patterns were also identified in other studies, although they were also named differently, e.g., “health conscious” instead of “prudent” [15]. Traditional patterns most closely reflect the cultural specificity of a country; for example, in a Dutch study, the “traditional” pattern was characterized by a high intake of potatoes, meat, and fat [15], while in French adults, it was based on the consumption of vegetables, vegetable fat, meat, and poultry [44]. The Chinese “traditional” pattern comprised pork, poultry, fish and prawns, eggs, fruit, dark-color vegetables, light-color vegetables, rice, water, yogurt, fungi, peanuts, sunflower seeds, and pastries [45]. On the other hand, the Polish “traditional” DP included white bread and bakery products, fried foods, cold meats, smoked sausages, hot dogs, and potatoes. In our study, this factor accounted for 11.4% of the total variance, while in the Dutch study, it accounted for −7.2% of the total variance [15], and as much as 17.5% in the Chinese study [45]. These differences may result from the specificity of culture, but also from its level of importance in conditioning current eating behaviors [46]. A higher percentage of explained variance could have been expected as older people are more attached to tradition. On the other hand, experiencing some health problems may favor a change in the eating habits of the elderly, and thus reduce the occurrence of the traditional dietary pattern.

The traditional dietary pattern can exhibit both beneficial and negative health characteristics. The “tradition47al” DP in Poland may be detrimental to health, due to the specificity of Polish cuisine, with its significant presence of fried foods, light flour products, potatoes, and fatty meats. Thus, the relatively low percentage of variance explained by this factor can be considered as positive. In the Portuguese study, the “traditional” DP identified with the “Mediterranean” DP (high consumption of vegetables, fruits, dairy, cereals/tubers, bread, fishery, and olive oil) was explained by as much as 59.1% of the variance in the elderly group [47], which confirms the attachment to tradition, and, at the same time, it informs us about the positive health effects resulting from the use of such a diet. In countries such as Portugal, but also Italy and Greece, the “Mediterranean” DP can be treated as both a traditional and a healthy pattern. In other countries, the pattern favorable to health was referred to as a “health conscious” DP, with a high intake of fruits, vegetables, poultry, fish, and alcohol [15], or as a “prudent” DP (i.e., high consumption of fruits, vegetables, lean meats, nuts, and seeds) [44,48]. In the Polish “prudent” DP, there were fruits and vegetables, but also buckwheat, oats, wholegrain pasta or other coarse-ground groats, fermented milk beverages, fresh cheese curd products, vegetable and fruit juices, and water. This factor accounted for 15.3% of the total variance, and thus explained more variance than other DPs, and also more variance than in other studies; for example, this factor accounted for 6.0% in the Dutch group [15], or 5.4% in women and 5.8% in men in the Quebec Longitudinal Study on Nutrition and Successful Aging [49].

Previous studies have shown that the differences in dietary patterns and their occurrence may be explained by the region of residence [18,20], both due to its cultural specificity and economic characteristics. In the study of Czarnocińska et al. [41], carried out in Polish young women, it was shown that women from more affluent regions represented the pattern characterized by a high consumption of vegetables and fruit more often, while a high consumption of fast food and sweets was characteristic of poorer regions. On the other hand, the presence of the “traditional” DP did not show any regional differences among young women. Our study showed no differences in the dietary patterns of elderly people from two regions with different GDP indexes. However, the lack of previous research carried out among older people in Poland makes it difficult to interpret the obtained results unambiguously. Despite the differences in GDP between the two regions, their territorial closeness (the southern part of Poland) could eliminate the effect of cultural differences, and, therefore, no relationship between the region and DPs has been demonstrated. In future research, attention should be paid to the search for other features of a region as potential factors to differentiate between both dietary patterns and food consumption in elderly people. The lack of regional differences in DPs may also suggest that their diversification in the elderly may result from factors that are more characteristic of the individual and their immediate environment, and may not relate to more global indicators.

Limitations in the functioning of the elderly, resulting from deteriorating health, but also from limited social contacts, are associated with disability in everyday activities [50], which may make it difficult for them to meet their own needs. It was confirmed that living with other people correlates with the quality of functioning of older people; for example, Ren and Treiman [51] showed that living only with one’s spouse was associated with less satisfaction with life and greater depression compared to living with adult children. In addition, previous studies have found an association between living alone and a poorer diet or increased nutritional risk [52]. Among other things, it was shown that people who lived alone consumed more food outside of their home, and skipped more meals than people who lived with their spouse [53]. They also ate meat, fish, seafood, raw vegetables, and legumes less frequently [54], and older women living alone tended to simplify the dining situation [55]. Nevertheless, in many studies, such a relationship has not been confirmed [34,35,36,53,54,55]. Our study showed no differences in the dietary patterns of people living alone and those living with a partner. However, such a relationship was confirmed for the group of people living with their family. It turned out that older people living with their family had greater chances of adherence to the “prudent” DP, both when the respondent was in a relationship or without a partner. Thus, the presence of adult children and grandchildren may favor a more adequate diet of the elderly. The study of Liu et al. [24] confirmed that people living with relatives consumed more food, and their diet was characterized by a higher quality and more correct frequency of eating meals. In our study group, the fewest people living with their family were in the upper tertile of the “traditional” DP, while most of these people were in the upper tertile of the “prudent” and “adverse” DPs, with the latter DP being mostly characteristic of the respondents who did not have a partner. This may mean that family members, not the elderly, are more important in food choices in these households [24], as evidenced by a smaller share of traditional food and a greater share of both recommended and non-recommended foods for health. Food decision-making processes may be dominated by those members of the household who have greater persuasion power, e.g., greater nutritional awareness that encourages care for the quality of the diet [56], but also special needs and preferences for food, e.g., children [23,57].

It is known that socioeconomic status (SES) is one of the factors that shows a strong relationship with health and diet in high-, middle- and low-income countries [58,59,60,61]. Some studies have confirmed the relationship of socioeconomic status with the identified dietary patterns [62,63], but also with higher scores on the healthy eating index and the Mediterranean diet score [63]. In middle- and low-income countries, a higher SES is associated with a more adequate diet, manifested by a higher consumption of fruit, vegetables, dairy products, and unprocessed meat [19,20,64,65,66,67]. Despite the fact that Poland is a middle–high income country [25], the obtained results did not confirm the relationship between high socioeconomic status and a healthy diet. It turned out that fewer respondents with a high SES represented the upper tertile of the “prudent” DP, but also the upper tertile of the “traditional” DP, compared to those with a low SES. In addition, their chances of being in the upper tertiles of these DPs were lower compared to those with a low SES (54% and 32%, respectively). On the other hand, more people with a high SES often ate food that was unfavorable to their health (the upper tertile of the “adverse” DP). These findings are confirmed by the results of some studies that have shown that in high-income countries, a higher SES was associated with better nutrition, while in middle-income countries, a higher SES was associated with both healthy and unhealthy dietary patterns [27,64,67]. The socio-economic development of these countries was conducive to changes in diet, leading to the transition from traditional diets to diets rich in fats and sugar [68]. This may explain the lower adherence to the “traditional” DP and greater adherence to the unhealthy pattern (the “adverse” DP) in the study group. Thus, the changes towards a healthy diet, mainly characteristic of highly developed countries, but also of middle-income countries, have not been confirmed in our sample [69]. Nevertheless, previous studies conducted in the Polish population showed that the elderly with a higher SES were characterized by a greater variety of food consumption, and a vaster range of healthy products, including fruit and vegetables, dairy and cereal products, fish, and fruit juices [70,71,72,73]. Thus, future research should investigate potential pathways through which the SES influences both food intake and adherence to dietary patterns in the elderly.

The identified differences in the dietary patterns of the elderly contribute to better understanding food intake, especially when it is related to people’s social situation. The study did not show differences in DPs between two regions; however, living with others and the SES showed relationships with DPs. A smaller share of traditional food, and a greater share of both recommended and non-recommended foods for health were observed when living with a family. Therefore, improving the diets of the elderly requires inter alia the involvement of younger people in learning about nutrition for the elderly. The study results can be used to support public policies and nutritional counseling among the elderly, but also among younger people living with the elderly. Interventions aimed at promoting healthier diets amongst the elderly should take account of social factors related to dietary patterns, which may mediate the effects of age-related factors that lead to health deterioration. However, future research is needed to more deeply recognize the factors and mechanisms that determine the diets of older people.

### Limitations of the Study

The data used for analysis were collected for the Polish population, and so they could not be generalized to other populations, especially those of different cultural backgrounds. Moreover, the KomPAN questionnaire used in the study was validated in the group of individuals who were up to 65 years of age. The cross-sectional design and collection of data at a single point in time did not permit conclusions to be drawn about causality. The use of a PCA to determine dietary patterns can be a methodological limitation of the study. The “a posteriori” nature of the patterns identified provides a realistic reflection of dietary patterns in our study population; however, the decisions on the extraction of the patterns from the PCA are, to some extent, subjective, and may affect the final dietary patterns that are analyzed. The three patterns identified in this study explain 32.9% of the overall variance, which is not a high rate; however, it is higher than in some other studies [15,74,75]. Another limitation of the study is that the sample was predominantly women, thus further exploration of the DPs in the male population would provide additional insights. The literature suggests that older men, especially those living alone, tend to have poorer cooking skills, associated with a lower quality of diet [76], and may be more affected by changes in their living situation.

## 5. Conclusions

Three dietary patterns were identified in the Polish elderly, i.e., the “traditional” DP, the “prudent” DP, and the “adverse” DP. The adherence to these dietary patterns was associated with socioeconomic status and living environment, i.e., alone, with a partner, or with family, while the region did not differentiate them. Less people living with their family were characterized by frequent consumption of traditional food, while more of them often consumed food typical for both “prudent” and “adverse” DPs. The presence of a respondent’s partner in the case of living with family did not differentiate the adherence to the DPs. Logistic regression models have shown that a high SES decreased the chances of adhering to the upper tertiles of the “prudent” and “traditional” DPs, while living with family increased the chances of adhering to both the upper and middle tertiles of the “prudent” DP. Identifying the dietary patterns of the elderly population contributes to better understanding the food intake of senior citizens living in different social situations, and can be used to support public policies and nutritional counseling among this age group. Interventions aimed at promoting healthier diets amongst the elderly should take account of underlying social factors that influence dietary patterns, which may mediate the effects of age-related factors that lead to health deterioration.

## Figures and Tables

**Table 1:** Study sample characteristics.

Variables	*N* = 437	(%)
Gender		
Female	289	66.1
Male	148	33.9
Age		
60–74 years old	331	75.7
75 years old or more	106	24.3
Place of residence		
Rural area	202	46.2
City ≤ 100,000 residents	89	20.4
City > 100,000 residents	146	33.4
Region		
Śląskie/Dolnośląskie	251	57.5
Świętokrzyskie	186	42.5
Household structure		
Living alone	67	15.3
Living with a partner	190	43.5
Living without a partner but with my family	61	14.0
Living with a partner and my family	119	27.2
SES index		
Low	145	33.2
Medium	154	35.2
High	138	31.6

**Table 2:** Factor-loading matrix for the dietary patterns identified using Varimax rotation.

Food Groups	Factor 1	Factor 2	Factor 3
	“Traditional” DP	“Prudent” DP	“Adverse” DP
White bread and bakery products, e.g., wheat bread, rye bread, wheat/rye bread, toast bread, bread rolls	0.638	−0.096	0.075
Fried foods (e.g., meat or flour-based foods, such as dumplings, pancakes, etc.)	0.555	−0.016	0.443
Cold meats, smoked sausages, hot dogs	0.595	0.100	0.225
Potatoes (excluding chips and crisps)	0.528	0.073	0.030
Buckwheat, oats, wholegrain pasta or other coarse-ground groats	−0.288	0.519	0.126
Fermented milk beverages, e.g., yoghurts, kefir (natural or flavoured)	−0.038	0.689	0.131
Fresh cheese curd products, e.g., cottage cheese, homogenized cheese, fromage frais	−0.012	0.593	0.022
Fruit	0.120	0.633	−0.296
Vegetables	0.185	0.660	−0.293
Vegetable juices or fruit and vegetable juice	−0.156	0.556	0.329
Water, e.g., mineral, tap water	−0.084	0.517	−0.106
Sweetened carbonated or still beverages, such as Coca-Cola, Pepsi, Sprite, Fanta, lemonade	0.095	−0.046	0.539
Energy drinks, such as Red Bull, Monster, Rockstar, or other	−0.033	0.055	0.585
Instant soups or ready-made soups, e.g., tinned, jar, concentrates (excluding frozen soup mixes)	0.195	0.004	0.619
Tinned (jar) meats	0.238	−0.016	0.643
Lard as a bread spread, or as an addition to meals/for frying/for baking, etc.	0.218	0.031	0.578
Vegetable oils or margarines or mixes of butter and margarines as a bread spread, or as an addition to meals/for frying/for baking	0.480	−0.074	0.080
Cheese (including processed cheese, blue cheese)	0.407	0.334	0.195
White meat, e.g., chicken, turkey, rabbit	0.423	0.391	0.138
Sweets, e.g., confectionary, biscuits, cakes, chocolate bars, cereal bars, other	0.496	−0.015	0.157
Wholemeal bread	−0.409	0.434	0.058
Milk (including flavoured milk, hot chocolate, latte)	0.203	0.457	0.186
Fish	−0.158	0.407	0.290
Eggs	0.096	0.429	0.229
Fruit juices	0.040	0.451	0.351
Fast foods, e.g., potato chips, hamburgers, pizza, hot dogs	−0.016	0.029	0.480
White rice, white pasta, fine-ground groats, e.g., semolina, couscous	0.182	0.303	0.231
Butter as a bread spread or as an addition to meals/for frying/for baking, etc.	0.345	0.106	0.035
Red meat, e.g., pork, beef, veal, mutton, lamb, game	0.353	0.063	0.316
Pulse-based foods, e.g., from beans, peas, soybeans, lentils	−0.336	0.356	0.139
Sweetened hot beverages, such as black tea, coffee, herbal or fruit teas	0.279	0.027	0.094
Tinned (jar) vegetables, e.g., pickles	0.157	0.258	0.315
Variance explained (%)	11.4	15.3	6.2
Total variance explained (%)	32.9		
Kaiser’s measure of sampling adequacy	0.798		

**Table 3:** Associations between dietary patterns and region, household status and SES index in the total sample (%).

Variables	“Traditional” DP ^a,^*			“Prudent” DP ^b,^*			“Adverse” DP ^c,^*		
	Bottom Tertile	Middle Tertile	Upper Tertile	Bottom Tertile	Middle Tertile	Upper Tertile	Bottom Tertile	Middle Tertile	Upper Tertile
Total sample *N* (%)	145 (33.2)	147 (33.6)	145 (33.2)	146 (33.4)	145 (33.2)	146 (33.4)	145 (33.2)	146 (33.4)	146 (33.4)
Region									
Śląskie/Dolnośląskie	29.9	36.7	33.4	33.1	33.5	33.4	32.7	35.5	31.8
Świętokrzyskie	37.6	29.6	32.8	33.9	32.8	33.3	33.9	30.6	35.5
Household structure									
Living alone	28.4	29.9	41.7	37.3	32.8	29.9	37.3	38.8	23.9
Living with a partner	30.5	35.8	33.7	37.4	35.3	27.3	32.6	32.1	35.3
Living without a partner, but with my family ^a,b,c,^*	36.1	36.1	27.8	31.1	29.5	39.4	29.5	34.4	36.1
Living with a partner and with my family ^a,b,c,^*	38.7	31.1	30.2	26.1	31.9	42.0	33.6	31.9	34.5
SES index									
Low	31.8	21.9	44.7	26.3	23.7	50.0	36.8	30.7	32.5
Medium	27.2	41.3	31.5	26.1	39.7	34.2	32.6	35.9	31.5
High ^a,b,c,^*	41.0	33.1	25.9	48.9	32.4	18.7	30.9	32.4	36.7
SES variables									
Self-reported financial situation									
Below average ^a,b,^*	4.5	8.3	8.8	6.5	7.8	8.3	10.3	11.7	9.6
Average	74.0	77.8	80.6	72.9	81.8	78.6	81.5	74.5	76.0
Above average ^a,b,c,^*	21.5	13.9	10.6	20.6	10.4	13.1	8.2	13.8	14.4
Family financial assistance									
No, although I have financial problems ^a,b,c,^*	8.9	10.3	5.4	11.0	8.8	6.9	11.0	10.3	6.8
Yes, because I have financial problems	6.4	7.6	2.7	9.0	6.2	4.8	8.2	7.6	11.0
There is no such need because my financial situation is satisfactory	76.7	71.0	83.7	75.2	74.7	80.0	75.3	72.4	77.4
Yes, although I have no financial problems ^a,b,c,^*	8.0	11.1	8.2	4.8	10.3	8.3	5.5	9.7	4.8
Social financial assistance									
No, although I have financial problems ^a,b,^*	12.1	15.2	5.4	15.2	6.9	8.9	20.5	14.5	14.4
Yes, because I have financial problems	1.6	3.4	0.0	1.4	2.1	1.4	1.4	1.4	2.1
There is no such need because my financial situation is satisfactory	85.6	80.0	93.9	82.8	90.3	89.0	77.4	82.1	83.5
Yes, although I have no financial problems ^c,^*	0.7	1.4	0.7	0.0	0.7	0.7	0.7	2.0	0.0
Education									
Primary ^a,b,c,^*	10.0	15.9	9.5	22.8	18.4	9.7	19.2	20.8	8.2
Vocational	34.7	38.6	40.8	26.2	28.1	33.8	43.8	29.7	37.5
Secondary	32.5	27.6	35.4	34.5	37.0	38.6	31.5	31.7	32.9
Higher education ^a,b,c,^*	22.8	17.9	14.3	30.1	16.5	17.9	5.5	17.8	21.4

^a^—statistical differences for the “traditional” DP; ^b^—statistical differences for the “prudent” DP, ^c^—statistical differences for the “adverse” DP; chi-square test, * *p* < 0.05.

**Table 4:** Associations between dietary patterns and region, household structures and SES index in the study sample (adjusted odds ratios with 95% confidence intervals).

Variables	“Traditional” DP		“Prudent” DP		“Adverse” DP	
	(Ref. Bottom Tertile)		(Ref. Bottom Tertile)		(Ref. Bottom Tertile)	
	Upper Tertile	*p*	Upper Tertile	*p*	Upper Tertile	*p*
Region (ref. Świętokrzyskie)	0.88 (0.70–1.11)	0.2898	0.99 (0.78–1.25)	0.9062	1.03 (0.82–1.31)	0.7626
Household structure						
Living with partner (ref. living alone)	0.74 (0.38–1.48)	0.4058	0.91 (0.46–1.83)	0.8014	1.68 (0.82–3.47)	0.1517
Living without a partner, but with my family (ref. living alone)	0.52 (0.22–1.26)	0.1413	1.58 (0.67–3.70)	0.0287	1.90 (0.78–4.69)	0.1515
Living with a partner and with my family (ref. living alone)	0.53 (0.25–1.11)	0.0883	2.02 (0.96–4.25)	0.0063	1.60 (0.74–3.46)	0.2268
SES index						
Medium SES (ref. low SES)	0.92 (0.70–1.24)	0.6130	0.83 (0.62–1.12)	0.2113	1.05 (0.79–1.40)	0.7496
High SES (ref. low SES)	0.68 (0.51–0.92)	0.0125	0.44 (0.33–0.62)	<0.0001	1.16 (0.86–1.57)	0.3314

*p*—significance level of the Wald’s test.

## Data Availability

Data presented in this study are available on request from the corresponding author.

## References

[1] Białecka-Dębek A., Pietruszka B. (2019). The association between hydration status and cognitive function among free-living elderly volunteers. Aging Clin. Exp. Res..

[2] Dean M., Raats M.M., Grunert K.G., Lumbers M. (2009). Factors influencing eating a varied diet in old age. Public Health Nutr..

[3] Von Ruesten A., Steffen A., Floegel A., van der A D.L., Masala G., Tjønneland A., Halkjaer J., Palli D., Wareham N.J., Loos R.J.F. (2011). Trend in obesity prevalence in European adult cohort populations during follow-up since 1996 and their predictions to 2015. PLoS ONE.

[4] Wądołowska L. (2010). Żywieniowe Podłoże Zagrożeń Zdrowia w Polsce.

[5] Michels K.B., Schulze M.B. (2005). Can dietary patterns help us detect diet–disease associations?. Nutr. Res. Rev..

[6] Kant A.K. (2010). Dietary patterns: Biomarkers and chronic disease risk. Appl. Physiol. Nutr. Metab..

[7] Wirfält E., Drake I., Wallström P. (2013). What do review papers conclude about food and dietary patterns?. Food Nutr. Res..

[8] Hu F.B. (2002). Dietary pattern analysis: A new direction in nutritional epidemiology. Curr. Opin. Lipidol..

[9] Tucker K.L. (2010). Dietary patterns, approaches, and multicultural perspective. Appl. Physiol. Nutr. Metab..

[10] Imamura F., Micha R., Khatibzadeh S., Fahimi S., Shi P., Powles J., Mozaffarian D. (2015). Dietary quality among men and women in 187 countries in 1990 and 2010: A systematic assessment. Lancet Glob. Health.

[11] Gubbels J.S., van Assema P., Kremers S.P. (2013). Physical activity, sedentary behavior, and dietary patterns among children. Curr. Nutri. Rep..

[12] Krusińska B., Hawrysz I., Słowińska M.A., Wądołowska L., Biernacki M., Czerwińska A., Gołota J.J. (2017). Dietary patterns and breast or lung cancer risk: A pooled analysis of 2 case-control studies in north-eastern Poland. Adv. Clin. Exp. Med..

[13] Bailey R.L., Mitchell D.C., Still C.D., Jensen G.L., Tucher K.L., Smiciklas-Wright H. (2007). A dietary screening questionnaire identifies dietary patterns in older adults. J. Nutr..

[14] Harrington J.M., Dahly D.L., Fitzgerald A.P., Gilthorpe M.S., Perry I.J. (2014). Capturing changes in dietary patterns among older adults: A lartent class analysis of on ageing Irish cohort. Public Health Nutr..

[15] De Jonge E.A.L., Rivadeneira F., Erler N.S., Hofman A., Uitterlinden A.G., Franco O.H., Kiefte-de Jong J.C. (2018). Dietary patterns in an elderly population and their relation with bone mineral density: The Rotterdam Study. Eur. J. Nutr..

[16] Gu Q., Sable C.M., Brooks-Wilson A., Murphy R.A. (2020). Dietary patterns in the healthy oldest old in the healthy aging study and the Canadian longitudinal study of aging: A cohort study. BMC Geriatr..

[17] Huang C.H., Martins B.A., Okada K., Matsushita E., Uno C., Satake S., Kuzuya M. (2021). A 3-year prospective cohort study of dietary patterns and frailty risk among comunity-dwelling older adults. Clin. Nutr..

[18] Seo A.R., Hwang T.Y. (2021). Relationship between dietary patterns and cardiovascular disease risk in Korean older adults. Int. J. Environ. Res. Public Health.

[19] Gaona-Pineda E.B., Martínez-Tapia B., Arango-Angarita A., En Sp M., Valenzuela-Bravo D., Gómez-Acosta L.M., Shamah-Levy T., Rodriguez-Ramirez S. (2018). Consumo de grupos de alimentos yfactores sociodemográficos en población mexicana. Salud Publica Mex..

[20] Pérez-Tepayo S., Rodriquez-Ramires S., Unar-Monguia M., Shamah-Levy T. (2020). Trends in the dietary patterns of Mexican adults by sociodemographic charakteristics. Nutr. J..

[21] Czarnocińska J., Jeżewska-Zychowicz M., Babicz-Zielińska E., Kowalkowska J., Wądołowska L. (2013). Postawy Względem Żywności, Żywienia i Zdrowia a Zachowania Żywieniowe Dziewcząt i Młodych Kobiet w Polsce.

[22] Wang J., Chen T., Han B. (2014). Does co-residence with adult children associate with better psychological well-beingd among the oldest old in China?. Aging Ment. Health.

[23] Rodrigues P.R.M., Monteiro L.S., Cunha D.B., Sichieri R., Pereira R.A. (2020). Adult food consumption by household composition: An analysis of the first National Dietary Survey, Brazil, 2008–2009. Public Health Nutr..

[24] Chang L., Fujin Y., Zhigang X., Xu T. (2021). Do living arrangements matter?—Evidence from eating behaviors of the elderly in rural China. J. Econ. Ageing.

[25] Miller V., Yusuf S., Chow C.K., Dehghan M., Corsi D.J., Lock K., Popkin B., Rangarajan S., Khatib R., Lear S.A. (2016). Availability, affordability, and consumption of fruits and vegetables in 18 countries across income levels: Findings from the prospective urban rural epidemiology (PURE) study. Lancet Glob. Health.

[26] Flores M., Macias N., Rivera M., Lozada A., Rivera-Dommarco J., Tucker K.L. (2010). Dietary patterns in Mexican adults are associated with risk of being overweight or obese. J. Nutr..

[27] Mayén A.L., Marques-Vidal P., Paccaud F., Bovet P., Stringhini S. (2014). Socioeconomic determinants of dietary patterns in low- and middle-income countries: A systematic review. Am. J. Clin. Nutr..

[28] Hinnig P.F., Monteiro J.S., de Assis M., Levy R.B., Peres M.A., Perazi F.M., Porporatti A.L., Canto G.L. (2018). Dietary patterns of children and adolescents from high, Medium and Low Human Development Countries and Associated Socioeconomic Factors: A Systematic Review. Nutrients.

[29] Andrade G.C., Da Costa Louzada M.L., Azeredo C.M., Ricardo C.Z., Martins A.P.B., Levy R.B. (2018). Out-of-home food consumers in Brazil: What do they eat?. Nutrients.

[30] Chen F., Short S.E. (2008). Household context and subjective well-being among the oldest old in China. J. Fam. Issues.

[31] Sun X., Lucas H., Meng Q., Zhang Y. (2011). Associations between living arrangements and health-related quality of life of urban elderly people: A study from China. Qual. Life Res..

[32] Quine S., Morrell S. (2007). Food insecurity in community-dselling older Australians. Public Health Nutr..

[33] Bae S., Urrutia-Rojas X., Patel D., Migala W.M., Rivers P.A., Singh K.P. (2007). Comparison of health behaviors among single-and multiple-member households. Am. J. Health Behav..

[34] Sheahan S.L., Fields B. (2008). Sodium dietary restriction knowledge, beliefs, and decision-making behavior of older females. J. Am. Acad. Nurse Pract..

[35] Hunter W., McNaughton S., Crawford D., Ball K. (2010). Does food planning mediate the association between living arrangements and fruit and vegetable consumption among women aged 40 years and older?. Appetite.

[36] Zhang J., Wu L. (2015). Cigarette smoking and alkohol consumption among Chinese older adults do living arrangements matter?. Int. J. Environ. Res. Public Health.

[37] GUS 2020 Wstępne Szacunki Produktu Krajowego Brutto w Przekroju Regionów 2019. file:///C:/Users/User/Downloads/wstepne_szacunki_pkb_w_przekroju_regionow_w_2019_r_2.pdf.

[38] (2014). Kwestionariusz do Badania Poglądów i Zwyczajów Zywieniowych Oraz Procedura Opracowania Danych (KomPAN^®^): Wersja Polskojęzyczna [Dietary Habits and Nutrition Beliefs Questionnaire and the Manual for Developing of Nutritional Data (KomPAN)].

[39] Kowalkowska J., Wadolowska L., Czarnocinska J., Człapka-Matyasik M., Galiński G., Jezewska-Zychowicz M., Bronkowska M., Dlugosz A., Loboda D., Wyka J. (2018). Reproducibility of a Questionnaire for Dietary Habits, Lifestyle and Nutrition Knowledge Assessment (KomPAN^®^) in Polish Adolescents and Adults. Nutrients.

[40] Wądołowska L., Kowalkowska J., Lonnie M., Czarnocinska J., Jeżewska-Zychowicz M., Babicz-Zielinska E. (2016). Associations between physical activity patterns and dietary patterns in a representative sample of Polish girls aged 13–21 years: A cross-sectional study (GEBaHealth Project). BMC Public Health.

[41] Czarnocińska J., Wądołowska L., Łonnie M., Kowalkowska J., Jeżewska-Zychowicz M., Babicz-Zielińska E. (2020). Regional and socioeconomic variations in dietary patterns in a representative sample of youn polish females: A cross-sectional study (GEBaHealth project). Nutr. J..

[42] Stanisz A. (2007). Przystępny kurs Statystyki. Tom 3. Analizy Wielowymiarowe.

[43] Milte C.M., McNaughton S.A. (2016). Dietary patterns and successful ageing: A systematic review. Eur. J. Nutr..

[44] Kesse-Guyot E., Andreeva V.A., Jeandel C., Ferry M., Herzberg S., Galan P. (2012). A healthy dietary pattern at midlife is associated with subsequent cognitive performance. J. Nutr..

[45] Sun J., Buys N., Shen S. (2013). Dietary patterns and cardiovascular disease-related risks in Chinese older adults. Front. Public Health.

[46] Kiviniemi M., Orom H., Giovino G.A. (2011). Race/ethnicity, psychological distress, and fruit/vegetable consumption. The nature of the distress-behavior relation differs by race/ethnicity. Appetite.

[47] Madeira T., Severo M., Oliveira A., Clara J.G., Lopes C. (2021). The association between dietary patterns and nutritional status in community-dwelling older adults—the PEN-3S study. Eur. J. Clin. Nutr..

[48] Parrott M.D., Shatenstein B., Ferland G., Payette H., Morais J.A., Belleville S., Kergoat M.J., Gaudreau P., Greenwood C.E. (2013). Relationship between diet quality and cognition depends on socioeconomic position in healthy older adults. J. Nutr..

[49] D’Amico D., Parrott M.D., Greenwood C.E., Ferland G., Gaudreau P., Belleville S., Laurin D., Anderson N.D., Kergoat M.J., Morais J.A. (2020). Sex differences in the relationship between dietary pattern adherence and cognitive function among older adults: Findings from the NuAge study. Nutr. J..

[50] Li L.W., Zhang J., Liang J. (2009). Health among the oldest-old in China: Which living arrangements make a difference?. Soc. Sci. Med..

[51] Ren Q., Treiman D.J. (2015). Living arrangements of the elderly in China and consequences for their emotional weel-being. Chin. Siciol. Rev..

[52] Wunderlich S., Brusca J., Johnson-Austin M., Bai Y., O’ Malley M. (2012). Eating behaviors of older adults participating in government-sponsored programs with different demographic backgrounds. Glob. J. Health Sci..

[53] Davis M.A., Murphy S.P., Neuhaus J.M. (1988). Living arrangements and eating behaviors of older adults in the United States. J. Gerontol..

[54] Larrieu S., Letenneur L., Berr C., Dartigues J.F., Ritchie K., Alpérovitch A., Tavernier B., Barberger-Gateau P. (2004). Sociodemographic differences in dietary habits in a population-based sample of elderly subjects: The 3C study. J. Nutr. Health Aging.

[55] Gustafsson K., Sidenvall B. (2002). Food-related health perceptions and food habits among older women. J. Adv. Nurs..

[56] Chen S.E., Liu J., Binkley J.K. (2012). An exploration of the relationship between income and eating behavior. Agric. Resour. Econ. Rev..

[57] Elstgeest L., Mishra G., Dobson A. (2012). Transitions in living arrangements are associated with changes in dietary patterns in young women. J. Nutr..

[58] Evans J.M., Newton R.W., Ruta D.A., MacDonald T.M., Morris A.D. (2000). Socioeconomic status, obesity and prevalence of type 1 and type 2 diabetes mellitus. Diabet. Med..

[59] Monteiro C.A., Moura E.C., Conde W.L., Popkin B.M. (2004). Socioeconomic status and obesity in adult populations of developing countries: A review. Bull. World Health Organ..

[60] Wagner K.H., Brath H. (2012). A global view on the development of non communicable diseases. Prev. Med..

[61] Dijkstra S.C., Neter J.E., Brouwer I.A., Huisman M., Visser M. (2014). Adherence to dietary guidelines for fruit, vegetables and fish among older Dutch adults; the role of education, income and job prestige. J. Nutr. Health Aging.

[62] Giuli C., Papa R., Mocchegiani E., Marcellini F. (2012). Dietary habits and ageing in a sample of italian older people. J. Nutr. Health Aging.

[63] Mullie P., Clarys P., Hulens M., Vansant G. (2010). Dietary patterns and socioeconomic position. Eur. J. Clin. Nutr..

[64] Giskes K., Avendano M., Brug J., Kunst A.E. (2010). A systematic review of studies on socioeconomic inequalities in dietary intakes associated with weight gain and overweight/obesity conducted among European adults. Obes. Rev..

[65] Aburto T.C., Pedraza L.S., Sánchez-Pimienta T.G., Batis C., Rivera J.A. (2016). Discretionary foods have a high contribution and fruit, vegetables, and legumes have alow contribution to the Total energy intake of the Mexican population. J. Nutr..

[66] Bruno-Fiscal C., Restrepo-Betancur L.F., Mendoza-Guerrero J.M. (2016). Supply of the main sources of energy, protein and fat in Mexico, 1961–2010. Rev. Esp. Nutr. Hum. Diet..

[67] Satheannoppakao W., Aekplakorn W., Pradipasen M. (2009). Fruit and vegetable consumption and its recommended intake associated with sociodemographic factors: Thailand National Health Examination Survey III. Public Health Nutr..

[68] Popkin B.M. (2004). The nutrition transition: An overview of world patterns of hange. Nutr. Rev..

[69] Seematter-Bagnoud L., Santos-Eggimann B., Nanchen D., Blanco J.M., Büla C., von Gunten A., Démonet J.F., Henchoz Y. (2021). Older People’s Health-Related Behaviors: Evidence from Three Cohorts of the Lc65+ Study. Behav. Med..

[70] Gutkowska K. (2002). Uwarunkowania wielkości i struktury spożycia żywności w gospodarstwach domowych osób starszych. Żyw. Człow. Metab..

[71] Kołłajtis-Dołowy A., Tyska M. (2004). Świadomość żywieniowa ludzi starszych w relacji do ich postaw i zachowań żywieniowych. Żyw. Człow. Metab..

[72] Waśkiewicz A., Sygnowska E. (2006). Wpływ poziomu wykształcenia na zachowania zdrowotne i czynniki żywieniowe związane z powstawaniem otyłości–badanie Pol-MONICA bis Warszawa. Zdr. Publ..

[73] Niedźwiedzka E., Wądołowska L. (2010). Analiza urozmaicenia spożycia żywności w kontekście statusu socjoekonomicznego polskich osób starszych. Probl. Hig. Epidemiol..

[74] Hardcastle A.C., Aucott L., Fraser W.D., Reid D.M., Macdonald H.M. (2011). Dietary patterns, bone resorption and bone mineral density in early post-menopausal Scottish women. Eur. J. Clin. Nutr..

[75] Shin S., Joung H. (2013). A dairy and fruit dietary pattern is associated with a reduced likelihood of osteoporosis in Korean postmenopausal women. Br. J. Nutr..

[76] Hughes G., Bennett K.M., Hetherington M.M. (2004). Old and alone: Barriers to healthy eating in older men living on their own. Appetite.

